# Understanding the evolution of immune genes in jawed vertebrates

**DOI:** 10.1111/jeb.14181

**Published:** 2023-05-31

**Authors:** Michal Vinkler, Steven R. Fiddaman, Martin Těšický, Emily A. O'Connor, Anna E. Savage, Tobias L. Lenz, Adrian L. Smith, Jim Kaufman, Daniel I. Bolnick, Charli S. Davies, Neira Dedić, Andrew S. Flies, M. Mercedes Gómez Samblás, Amberleigh E. Henschen, Karel Novák, Gemma Palomar, Nynke Raven, Kalifa Samaké, Joel Slade, Nithya Kuttiyarthu Veetil, Eleni Voukali, Jacob Höglund, David S. Richardson, Helena Westerdahl

**Affiliations:** ^1^ Department of Zoology Faculty of Science Charles University Prague Czech Republic; ^2^ Department of Biology University of Oxford Oxford UK; ^3^ Department of Biology Lund University Lund Sweden; ^4^ Department of Biology University of Central Florida Florida Orlando USA; ^5^ Research Unit for Evolutionary Immunogenomics Department of Biology University of Hamburg Hamburg Germany; ^6^ Institute for Immunology and Infection Research University of Edinburgh Edinburgh UK; ^7^ Department of Veterinary Medicine University of Cambridge Cambridge UK; ^8^ Department of Ecology and Evolutionary Biology University of Connecticut Storrs Connecticut USA; ^9^ School of Biological Sciences University of East Anglia Norwich UK; ^10^ Department of Botany and Zoology Masaryk University Brno Czech Republic; ^11^ Menzies Institute for Medical Research University of Tasmania Hobart Tasmania Australia; ^12^ Department of Parasitology University of Granada Granada Spain; ^13^ Department of Biological Sciences University of Memphis Memphis Tennessee USA; ^14^ Department of Genetics and Breeding Institute of Animal Science Prague Uhříněves Czech Republic; ^15^ Faculty of Biology Institute of Environmental Sciences Jagiellonian University Kraków Poland; ^16^ Department of Science Engineering and Build Environment Deakin University Victoria Waurn Ponds Australia; ^17^ Department of Genetics and Microbiology Faculty of Science Charles University Prague Czech Republic; ^18^ Department of Biology California State University Fresno California USA; ^19^ Department of Ecology and Genetics Uppsala Universitet Uppsala Sweden

**Keywords:** adaptation, adaptive immunity, evolutionary immunology, genomics, host‐parasite interactions, immunogenetics, innate immunity, MHC, molecular evolution, vertebrates

## Abstract

Driven by co‐evolution with pathogens, host immunity continuously adapts to optimize defence against pathogens within a given environment. Recent advances in genetics, genomics and transcriptomics have enabled a more detailed investigation into how immunogenetic variation shapes the diversity of immune responses seen across domestic and wild animal species. However, a deeper understanding of the diverse molecular mechanisms that shape immunity within and among species is still needed to gain insight into—and generate evolutionary hypotheses on—the ultimate drivers of immunological differences. Here, we discuss current advances in our understanding of molecular evolution underpinning jawed vertebrate immunity. First, we introduce the immunome concept, a framework for characterizing genes involved in immune defence from a comparative perspective, then we outline how immune genes of interest can be identified. Second, we focus on how different selection modes are observed acting across groups of immune genes and propose hypotheses to explain these differences. We then provide an overview of the approaches used so far to study the evolutionary heterogeneity of immune genes on macro and microevolutionary scales. Finally, we discuss some of the current evidence as to how specific pathogens affect the evolution of different groups of immune genes. This review results from the collective discussion on the current key challenges in evolutionary immunology conducted at the ESEB 2021 Online Satellite Symposium: Molecular evolution of the vertebrate immune system, from the lab to natural populations.

## Graphical abstract

Reviewing current advances in our understanding of molecular evolution underpinning vertebrate immunity, we propose hypotheses to explain differences in selection modes across immune genes and discuss supporting evidence.

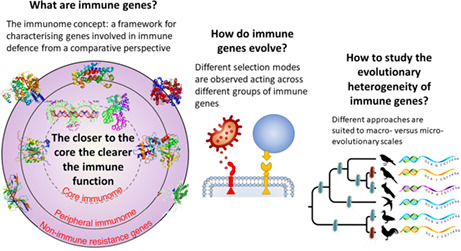

## 1 INTRODUCTION

Evolutionary immunology represents an important branch of infection biology that synergizes immunology and evolutionary studies across diverse taxa (Vinkler et al., 2022). Using the conceptual framework of evolutionary biology to research biomedically relevant issues (Stearns & Koella, 2008), evolutionary immunology addresses fundamental questions on the origins and consequences of inter‐ and intraspecific variation in immunity and how these have resulted in variation in disease resistance. Based on comparative approaches, it aims to provide a foundation for understanding the diversity of evolutionary adaptations seen across immunity. However, to meet this aim, extensive development in the field is still needed. The central focus in evolutionary immunology is to understand host adaptations to either combat or tolerate pathogens. Such adaptation is context dependent: pathogenic microorganisms are only part of the immunobiome (the set of organisms—including commensal and mutualist symbionts—that can live in or on a host; Horrocks et al., 2011). The complex interactions between hosts and these co‐evolving organisms create variation in the selective pressures acting on host immune systems, ultimately leading to the emergence of extensive diversity in immune defence strategies (Buchmann, 2014; Danilova, 2006). Additionally, life history variation and evolutionary history, including genetic background, can constrain immunogenomic variation due to trade‐offs and pleiotropy (Norris & Evans, 2000; Schwenke et al., 2016), generating complex selective landscapes beyond those driven only by pathogens.

While classical model species are invaluable for gaining insight into mechanistic features of the complex jawed vertebrate immune system (Mestas & Hughes, 2004), comparative research is needed to reveal functional immunological variation at both the inter‐ and intraspecific level. Unlike most morphological and physiological systems, where traits may show relatively straightforward adaptations, immunity evolves as a regulatory network integrating numerous independently acting defence cells and mechanisms to control pathogens. Through arms races consistent with Red Queen dynamics (Woolhouse et al., 2002), host–pathogen coevolution in favour of the host leads to optimal pathogen control, or minimal host damage, through the complex regulation of the immune system (Ashley et al., 2012). As a consequence, in certain cases, adaptation to pathogens does not lead to increased disease resistance but, instead, promotes tolerance to infection (resilience) (Råberg et al., 2007).

Immune genes are among the most rapidly evolving gene classes within animals, with remarkable levels of polymorphism maintained in certain immune genes, even beyond speciation events (Bustamante et al., 2005; Figueroa et al., 1988; Fumagalli et al., 2011; Hillier et al., 2004; Lenz et al., 2013; Těšický & Vinkler, 2015). Guided by these observations, intensive evolutionary research focusing on the Major Histocompatibility Complex (MHC) has occurred during the past decades (reviewed in Apanius et al., 1997; Kaufman, 2018; O'Connor et al., 2019; Radwan et al., 2020; Sommer, 2005; Spurgin & Richardson, 2010). However, it has become clear that while the MHC is essential for host adaptation to diseases, it alone is not enough to explain variation in host resistance to infection (Acevedo‐Whitehouse & Cunningham, 2006). Consequently, other immune genes have started to receive considerable attention, including pattern‐recognition receptors (PRRs) (Alcaide & Edwards, 2011; Davies et al., 2021; Krchlíková et al., 2021; Smirnova et al., 2000; Tschirren et al., 2012; Velová et al., 2018; Vinkler & Albrecht, 2009), antimicrobial peptides (AMPs) (Chapman et al., 2016) and signalling pathway components (Downing et al., 2010; Hyland et al., 2021).

About 10% of genes in the jawed vertebrate genomes are currently considered as involved in immune function (the Gene Ontology annotation category ‘immune system process’, filtered on 2021‐08‐27 based on Uniprot IDs, human: 2857 genes, mouse: 1586, chicken: 1576). The type and strength of selection that act on these genes may vary considerably. Multiple evolutionary forces modulate immunogenetic diversity and a key current challenge is to understand how these different evolutionary forces and resulting mechanisms influence the diverse array of immune genes in vertebrate genomes. Recent advances in immunogenomics provide methods to describe this variation (Holt, 2015), but the availability of genomic data alone does not provide all the insight needed. To further our understanding, we need to formulate detailed hypotheses and testable predictions that can explain this immunogenetic diversity. Developing such a scientific framework requires collaboration between researchers with diverse expertise and methodological backgrounds, interlinking evolutionary biology and eco‐immunology with classical immunology and pathogen biology at molecular and cellular levels (Seed, 1993; Adamo, 2004; Vinkler & Albrecht, 2011). We used the ESEB 2021 Satellite Symposium: ‘Molecular evolution of the vertebrate immune system, from the lab to natural populations’ to initiate an interdisciplinary discussion into the molecular evolution of the vertebrate immune system. Based on the ground‐breaking research presented, and discussions throughout this symposium, we review some of the key current concepts and advances in evolutionary immunology. We hope to collectively contribute to a conceptual framework that can better explain the evolution of the immunogenetic diversity observed in jawed vertebrates.

## 2 WHAT ARE IMMUNE GENES?

Hosts use a very broad array of defences against pathogens (microorganisms and multicellular parasites causing disease to their host) including both classical immune mechanisms as well as non‐immune defences, for example, behavioural strategies to avoid infection, physical barriers and general physiological processes (Schmid‐Hempel, 2011). To rationally study the evolution of the immune system, a definition of what an *immune gene* is must be considered. Is an immune gene one which is expressed in immune cells and tissues? Or a gene whose expression is modulated in response to an infection? Or only one where the gene product directly interacts with a pathogen? Answering these questions is not easy, given that roughly two thirds of the vertebrate genome is active in at least one immune cell type (Heng et al., 2008). We may call the entire genetic basis of immunity the ‘immunome’, a term originally coined to describe the totality of rearranged antigen‐binding capability in the adaptive immune system of all individuals of a species (Pederson, 1999). More recently this term has been adopted to conform to other widely used ‘‐omes’ to mean all immunology‐related genes in the genome (Ortutay et al., 2007). However, a precise definition as to which specific genes are included is still lacking, and this problem is further compounded when we consider that immune genes may differ across animal taxa.

**FIGURE 1:**
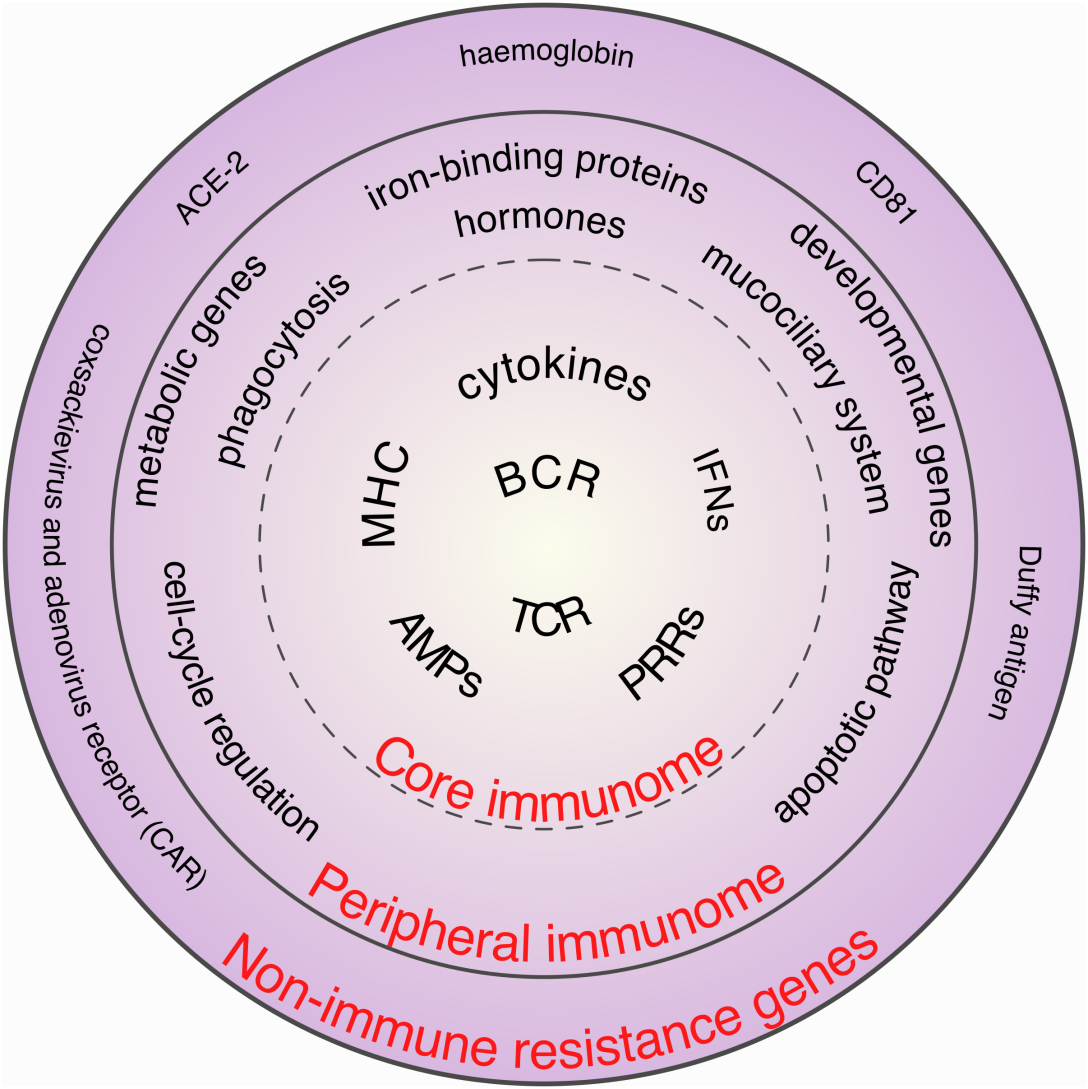
A proposed system for the hierarchical classification of immune genes. A gene considered relevant to the immune system will fall into one of three categories: (i) *the core immunome*, (ii) *the peripheral immunome*, and (iii) *non‐immune resistance genes*. Core immunome components are unequivocally (and in many cases, exclusively) related to immune function. Peripheral immune genes contribute to aspects of the immune response, despite also having additional roles in the physiological functioning of other body systems. Non‐immune resistance genes (NIRGs) are genes that do not form part of the immune system but can functionally confer resistance to a pathogen and thus are important to consider as part of an immune phenotype. Examples of such genes (or gene classes/families) are given in each compartment. The dashed line between core and peripheral immune components indicates that there is likely a continuum of genes whose function ranges from exclusively immune to almost entirely for another physiological function, and that some genes may lie ambiguously between the two categories. Abbreviations: ACE‐2, angiotensin converting enzyme‐2; MHC, major histocompatibility complex; AMPs, anti‐microbial peptides; TCR, T‐cell receptor; BCR, B‐cell receptor; IFNs, interferons; PRRs, pattern‐recognition receptors.

To provide a framework upon which immune genes can be discussed, we propose a hierarchical set of definitions that relate to three layers of the ‘immunome’ (Figure 1). (i) the *core immunome*: genes whose primary (and in many cases, only) physiological function is in the recognition of, and/or response to, pathogens; (ii) the *peripheral immunome*: genes with a clear immunological role, but which also contribute to non‐immune physiological function and (iii) non‐immune resistance genes (NIRGs; which could be called the *accessory immunome* or even the *resistome*). However, Beutler et al. (2005) proposed a broader definition for this last category, that is, genes (or alleles of genes) that are not normally involved in immunity, but are nevertheless associated with resistance to a particular (class of) pathogen because they become targets for pathogen molecules or can interfere with some phase of infection. Therefore, NIRGs functionally contribute to immunity in the sense that they affect the interaction between pathogen and host, but often the resistance‐conferring alleles of NIRGs are highly pathogen‐specific.

*Core immunome* genes are undeniably part of the immune system. Examples include genes required for the recognition of pathogens (e.g. PRRs, T‐cell receptor [TCR] and B‐cell receptor [BCR] genes), genes involved in the antigen presentation pathways (e.g. MHC class I and II), or genes involved in anti‐pathogen responses (e.g. AMPs and interferons). However, for many genes, involvement in the immune response may be less obvious, or part of a wider suite of physiological activities. These *peripheral immunome* genes could include, for example, those that code for iron‐binding proteins, such as transferrin which plays a key role in iron homeostasis. While this role may, at first glance, seem far removed from immunity, transferrin is important to sequester iron away from blood‐borne pathogens and thus acts as an anti‐microbial protein (Sridhar et al., 2000; Watanabe et al., 1997). Similarly, the apoptotic (controlled cell death) pathway is central to development and in responses to cellular stress but is also involved in immunity (Jorgensen et al., 2017). This is perhaps exemplified best by the efforts pathogens make to subvert host cell death pathways (Robinson & Aw, 2016). Further pleiotropy can be observed in developmental genes such as the Notch signalling pathway (Laky & Fowlkes, 2008), which has a role in both embryogenesis and the function of immune cells (Radtke et al., 2010), and transforming growth factor beta, which is involved in regulating immune responses but also has several other physiological roles (Travis & Sheppard, 2014).

The third group of genes, NIRGs, is less clearly defined but forms part of a host's resistance phenotype to pathogens. These NIRGs would not typically be considered part of the immune system per se but are genes whose product variants can interfere with pathogen cell entry and other pathogen life‐cycle processes. A classic example is the haemoglobin genes, where some alleles (sickle cell anaemia and thalassaemia) confer protection against malaria and are maintained in the human population despite considerable fitness costs to the host (reviewed in Luzzatto, 2012). Alleles encoding variants of the host cell receptors usually used by pathogens to enter a cell may confer resistance to infection. For example, the partial deletion allele of the chemokine receptor CCR5, alleles of CD81 and alleles of human angiotensin‐converting enzyme (ACE)‐2 provide resistance to human immunodeficiency virus (HIV), hepatitis C virus (HCV) and SARS‐CoV‐2, respectively (McLaren et al., 2015; Sun et al., 2015; Suryamohan et al., 2021). Indeed, interspecies sequence variation in NIRGs is likely to contribute to host range specificity in pathogens (Clark et al., 2022; Wei et al., 2021).

## 3 RETRIEVING IMMUNE GENE SETS FROM PUBLIC REPOSITORIES

When working with genomic data (BOX 1), immune gene lists are usually generated based on annotations obtained from public repositories. Current genome browsers, such as Ensembl (Cunningham et al., 2022), offer curated annotations (derived from, e.g. UniProtKB/Swiss‐Prot; UniProt Consortium, 2019) as well as unreviewed automatic annotations for proteins translated from the nucleotide sequences deposited in the International Nucleotide Sequence Database Consortium (INSDC) synchronizing European Nucleotide Archive (ENA), National Center for Biotechnology Information (NCBI) GenBank and DNA Data Bank of Japan (DDBJ). These universal annotations are based on generic structural, expression and functional gene characteristics. Among the most commonly used ones are the taxon‐specific annotations provided by the Kyoto Encyclopedia of Genes and Genomes (KEGG; https://www.genome.jp/kegg/; Kanehisa & Goto, 2000) and the Gene Ontology (GO) term database (entry e.g. via AmiGO; http://amigo.geneontology.org/amigo; Carbon et al., 2009). While widely used in evolutionary genomics (see e.g. Feigin et al., 2018; Figueiró et al., 2017; Huang et al., 2017; Khan et al., 2019; Shultz & Sackton, 2019), the KEGG pathways are available for specific immune responses, grouping all genes revealed as differentially expressed in annotation studies even when they do not directly contribute to immunity. Similarly, the general GO terms (e.g. immune system process: GO: 0002376) define wide ranges of immune response‐related genes from the core immunome to NIRGs, some of which are only weakly associated with the actual immune defence.

BOX 1 **Studying the functional consequences of immune gene variation**

High‐throughput sequencing allows for powerful comparative analyses of immune genes. Alignment of protein sequences, for a species of interest against human and/or other model organisms, can quickly lead to hypotheses about conserved function. To circumvent the lack of reference genomes and knowledge of splicing variants (i.e. isoforms) in many non‐model species, de novo transcriptome assemblies can be used to identify protein‐coding sequences, which can then be aligned against validated sequences from well‐characterized species (Flies et al., 2017; Sun et al., 2012).

While Ensembl serves as the tool for initial gene analysis (Cunningham et al., 2022), UniProt remains a first‐stop resource for understanding functional domains of proteins (UniProt Consortium, 2019). Key functional regions can also be interrogated using publicly available software programs. For example, the Eukaryotic Linear Motif resource can predict functional motifs associated with signal transduction, protein trafficking and protein binding domains (Dinkel et al., 2014). These analyses can be further expanded by the application of in silico modelling to reveal variations in protein physicochemical properties (Follin et al., 2013; Těšický et al., 2020). In addition, machine learning technology such as AlphaFold is fundamentally changing our ability to predict protein structure (Jumper et al., 2021). Validation of the predicted structure and function of proteins can then be done via the expression of recombinant proteins (see below).

Signatures of selection in protein‐coding DNA can be detected by the rate of non‐synonymous to synonymous substitutions (*dN*/*dS* method; reviewed in Sackton, 2020). Prior to any selection analysis, recombination should be assessed (e.g. by GARD; Kosakovsky Pond et al., 2006), especially when working with closely related species or population data. If it is found, positive selection should be tested separately across different non‐recombined DNA fragments. Several popular packages incorporating multiple methods to detect selection have been developed, such as paml (with CODEML models; Yang, 2007) or Hyphy package (Kosakovsky Pond et al., 2005), embedded within a user‐friendly Datamonkey webpage interface (https://www.datamonkey.org/analyses). The standard codon‐based models can be divided into three classes differing in assumptions about ω variation among branches and sites (Hölzer & Marz, 2021): (i) Site models assuming that ω can vary across sites (CODEML or FUBAR, REL or FEL from Hyphy; Kosakovsky et al., 2005; Murrell et al., 2013) detect long‐lasting pervasive selection. (ii) Branch‐site models assume that ω can vary not only across sites but also among lineages and detect episodic selection (MEME, BUSTED, aBS‐REL or CODEML; Murrell et al., 2012, 2015; Smith et al., 2015). (iii) To test for lineage‐specific rate of evolution, branch models may assume that selection varies across lineages while keeping constant ω estimates across whole genes. Recently, to automate positive selection scans in large‐scale datasets, comprehensive pipelines, such as Poseidon (Hölzer & Marz, 2021), or highly customizable DIGGN pipeline (Picard et al., 2020) requiring only nucleotide sequences as an input and performing all steps (alignment and its cleaning, gene tree reconstruction, recombination and positive selection screening) have been developed. As some methods might be prone to false positive results (Nozawa et al., 2009), and results from different methods do not always correspond (Areal et al., 2011), comparing results from multiple methods is always beneficial (Těšický et al., 2020). Furthermore, codon‐usage analysis can be used to explore convergent evolution among species and distinguish it from trans‐species polymorphism (Lenz et al., 2013).

To reveal the actual immunological effect of the adaptive variation, functional testing is necessary. Recombinant protein production has seen major advances and new proteins can be produced in a few weeks once the coding sequence is determined. DNA coding sequences can be amplified via PCR from cDNA or ordered synthetically from commercial suppliers. Plasmid DNA vectors for prokaryotic and eukaryotic expression systems can be used, where DNA is inserted into plasmid expression vectors via restriction digest and ligation, or via methods with improved flexibility such as Gibson assembly (Gibson et al., 2009). Studies using recombinant proteins, for example, have helped to reveal functional variation in mammalian and avian *TLRs* and validate bioinformatic predictions (Fiddaman et al., 2022; Keestra & van Putten, 2008; Levy et al., 2020; Walsh et al., 2008).

Expression vectors can be also used to produce soluble recombinant proteins for functional assays. Most cytokines are readily secreted from eukaryotic cell lines and can be used directly from supernatant or in a purified form in functional assays. For example, IFNG should upregulate MHC class I on host cells and IL2 should drive the activation and proliferation of T cells. Nevertheless, possible interspecific variation in the immunological activity of various regulators needs to be anticipated, as documented, for example, by the variation in IFNG regulation of NO signalling in mammals (Bilham et al., 2017). The interaction of surface proteins can be tested using fluorescent fusion proteins that allow binding interactions to be rapidly determined via fluorescent microscopy or flow cytometry (Flies et al., 2020). Additionally, the production of recombinant MHC proteins has been used to identify key pathogen peptides that can be used to understand disease pathology and support vaccine development (Halabi et al., 2021; Wang, Yue, et al., 2021).

Highly conserved structures can often be tested via cross‐species binding assays. For example, the amino acids of the receptor binding domains (MYPPPY) and protein trafficking domains (YVKM) of key T cell co‐signalling proteins CD28 and CTLA4 are conserved across mammals, birds, reptiles and amphibians. Development of additional expression vectors that swap amino acids, such as changing tyrosines to alanines in the protein trafficking domain (YVKM to AVKM) can be used in function assays to validate structure and function (Wong et al., 2021).

Final validation of protein structure and interactions can be done through protein crystallization studies. This has previously been out of range for most evolutionary immunologists and ecologists, limiting this application to variation between model organisms (Halabi & Kaufman, 2022; Koch et al., 2007). However, structural biology studies have begun showing interest in non‐model species and making exciting discoveries. For example, a novel type of TCR has been recently discovered in marsupials (Morrissey et al., 2021). Although most of the above‐mentioned approaches have been applied to few species so far, wider application of these methodological improvements could level the field for evolutionary immunology in terms of resource availability and knowledge gaps.

The use of immune gene databases will differ depending on the specific research question and study organism. While some databases provide only simplistic immune gene lists with assignments of genes in functional categories (e.g. ImmPort for human genes, where a user must retrieve all other information from third‐party databases, Bhattacharya et al., 2018), other databases are more comprehensive (AmiGO, Reactome, Gillespie et al., 2022; KEGG and InnateDB, Breuer et al., 2013) enabling retrieval of additional information (e.g. gene function, cellular localisation and alternative gene identifiers (IDs), thus allowing easy crosslinking with other specialized databases—Uniprot, PDB, etc.). Gene annotations in these databases are only properly curated for a few selected species, namely humans or mice, whereas most annotations (even for some model species) rely on automatic annotations. While it might be less problematic for core immune genes whose function is likely to be well conserved across jawed vertebrates, caution is needed when considering genes of the peripheral immunome or NIRGs. Unfortunately, these different categories of immune genes cannot be automatically retrieved from the current immune databases, which would be convenient when working with large datasets. On the other hand, this problem could be partially overcome by focusing on the overlap of immune gene lists between several species or several immune gene databases. Unlike core immune genes, peripheral immune genes and (especially) NIRGs are less likely to overlap between species and/or databases. To this end, continued endeavour to create more specialized immune databases—such as the Avian Immunome Database (AVIMM, Mueller et al., 2020), the Immunome Database for Marsupials and Monotremes (IDMM, Wong et al., 2011) and the porcine immunome (Dawson et al., 2013)—may increase the reliability with which immunome genes can be identified. Nonetheless, the problem with these resources is that their maintenance is demanding, and they may end up out of date. In addition, these databases have been curated using various levels of inclusivity and detection methods, such as literature searches, gene ontology terms, conserved protein domains, orthology searches or a combination of approaches (e.g. Wong et al., 2011) and, therefore, differ in content.

Although current immune gene databases allow the filtering of immune gene sets based on multiple criteria, such as molecular function, as far as we know, their vocabularies do not allow easy extraction of general immune gene categories (such as surface receptors, adaptor molecules, signalling molecules, etc.). Rather, they group immune genes into hierarchical functionally interlinked pathways being involved in a particular molecular mechanism, for example, in MHC class I antigen presentation or MyD88‐dependent pathways. Thus, they group together many functionally unrelated genes ranging from surface receptors to transcription factors. Clustering genes in this way is often subject‐specific, hindering direct comparison among the multiple terms. Depending on the research question, manual curation of such immune gene categories might be necessary but may be unfeasible in large‐scale evolutionary studies. In addition, in the case of manual curation, some commonly used analyses such as gene set enrichment analysis (Subramanian et al., 2005) may be difficult to apply.

Using gene annotation based on orthology inference is another aspect that requires particular caution. The level of true orthology assignment decreases with increasing genetic distance (Gabaldón & Koonin, 2013). In such cases, the orthology relationship can be inferred from multiple species and then compared, for example, as in Shultz and Sackton (2019). While for most immune genes one‐to‐one (one2one) ortholog relationships prevail (Těšický et. al, unpublished), caution is needed in dynamically evolving multi‐gene families, for example, in MHC genes, chemokines or beta‐defensins (Bean & Lowenthal, 2022; Machado & Ottolini, 2015; Nei & Rooney, 2005; Nomiyama et al., 2013) with more complicated relationships, such as one2many or many2many orthology. This issue is compounded by a lack of harmonization of gene names and their identifiers between different species and/or different databases. For instance, in different databases different gene symbols are given to the same gene, or the same gene symbol is given to different but similar genes (see e.g. AVIMM database, Mueller et al., 2020, and gProfiler, Reimand et al., 2007). Given these limitations of gene classification systems, it remains critically important for a given study to consider both species‐specific immune processes and potential selective forces that might be acting on the ‘genes of interest’.

## 4 HALLMARKS OF IMMUNE GENE EVOLUTION

Given their crucial role in rapid host–pathogen coevolution, immune genes (especially the core immunome, Figure 1) have been hypothesised to undergo more dynamic evolution than other gene groups, leading to high levels of diversification—interspecific as well as intraspecific variation (Woolhouse et al., 2002). This has been confirmed in genomic studies of humans and other organisms (Ekblom et al., 2010; Hillier et al., 2004; Nielsen et al., 2005; Sackton et al., 2007). Several underlying evolutionary mechanisms have been suggested to increase and maintain this immunogenetic variability. Diversity in host immune genes increases through repeated episodes of pathogen‐driven selective sweeps (as part of a reciprocal coevolutionary arms race) when the direction of positive selection differs between species, between and within populations and between generations. Adaptations usually involve only short functional parts of the genes, such as peptide‐binding region in MHC or few functional single‐nucleotide polymorphic sites (SNPs) around the PRR ligand‐binding region (Hughes & Nei, 1988; Lenz et al., 2013; Velová et al., 2018), with rest of the sequence being functionally constrained and under negative selection.

Within populations balancing selection can maintain high polymorphism through heterozygote advantage, negative frequency‐dependent selection or fluctuating selection (Klein & Ohuigin, 1994; Minias & Vinkler, 2022; Spurgin & Richardson, 2010; Vinkler et al., 2022; Westerdahl et al., 2004; Woolhouse et al., 2002). None of these three mechanisms are mutually exclusive and they may also interact with sexual selection to maintain high polymorphism (Ejsmond et al., 2014). However, even alleles that are maintained for long time periods by balancing selection initially diversify through reciprocal adaptations to specific pathogens driven by positive selection (Těšický & Vinkler, 2015). Moreover, immune gene polymorphism within a species may also increase through introgression, resulting from hybridization between closely related species (Hedrick, 2013). Despite increasing evidence of introgression in different taxa and immune gene types (Fijarczyk et al., 2018; Grossen et al., 2014; Jagoda et al., 2018; Nadachowska‐Brzyska et al., 2012), how common this mechanism is remains unclear. This is, at least partly, because introgressed alleles can subsequently be maintained by balancing selection, complicating our ability to distinguish them from shared standing variation (trans‐species polymorphism; Těšický & Vinkler, 2015).

Genes that belong to the core immunome have frequently been investigated also for other important evolutionary phenomena, such as convergent (Storz, 2016; Yeager & Hughes, 1999) and parallel/concerted evolution (Pavlovich et al., 2018; Świderská et al., 2018; Těšický et al., 2020; Wutzler et al., 2012), allowing independent evolution of functionally related immune variants, or gene conversion (Högstrand & Böhme, 1999; Huang et al., 2011; Velová et al., 2018; Yeager & Hughes, 1999), homogenizing sequences across gene loci. While these phenomena could play a role in immune gene evolution more frequently than in other gene groups, such patterns are typically observed only in specific functional domains or structural motives (Hughes & Nei, 1988, 1989; Smirnova et al., 2000; Velová et al., 2018) and, since evolution is stochastic, evolve non‐universally only in specific taxa (Vlček et al., 2022). Future systematic research will be needed to reveal frequency of their occurrence in vertebrates.

We can also ask if there are any hallmarks of molecular adaptation that differ between traditionally recognized adaptive immune genes and innate immune genes. Such differences can be expected if all parts of innate immunity are ancient (e.g. billions of years old), as has been argued, while adaptive immunity (at least TCR/BCR‐based) emerged relatively recently in jawed vertebrates (~450 million years ago; Marchalonis et al., 2002). However, it is important to stress that innate and adaptive immunity do not form distinct functional systems, but rather refer to interlinked layers of regulatory and effector mechanisms that are defined based on the principal mechanism of the origin of their receptor variation: germ‐line encoded (innate) versus somatically rearranged (adaptive: BCR and TCR). Clonal somatic rearrangement in BCR/TCR allows B‐ and T‐cells to detect an enormous variety of antigens, making these receptors functionally unique. Throughout the evolution of the immune system, the original defence mechanisms have been supplemented, but typically not replaced by new ones (Danilova, 2006; Marchalonis et al., 2002). Thus, highly redundant, carefully regulated, immune mechanisms emerged in vertebrate hosts, crosslinking the two broad sections of immunity.

The most conspicuous hallmark of many immune genes is their rapid evolution. This involves high interspecific variation, that is, diversification of genes among species, as well as high polymorphism, that is, the maintenance of many alleles per gene within a species. While molecular variability is particularly marked in the adaptive immune genes, as seen with MHC genes (Piertney & Oliver, 2006; Schlesinger et al., 2014), single‐copy innate immune genes have been suggested to be rather conserved (e.g. Eisen & Chakraborty, 2010; Roach et al., 2005) and even invariant at a microevolutionary scale (although multigene families of innate immune genes can be quite diverse, see BOX 2). Yet, adaptive immunity, understood as a pathway, groups many genes with diverse functional roles, including, for example, co‐receptors and cytokines regulating T‐cell and B‐cell development. As adaptive immune genes beyond the MHC are rarely investigated, we do not know if these components also exhibit signatures of increased adaptive variability. In parallel, the immunological mechanisms grouped under the term ‘innate immunity’ often have little in common except for being dependent on germline‐encoded recognition of microbes or host damage. It has recently been shown that while some innate immune genes appear truly conserved, for example, some AMPs (Chapman et al., 2016), others exhibit reasonably high inter‐ and intraspecific variation as well as signatures of positive selection (Vivier & Malissen, 2005), for example, some other AMPs (Lazzaro et al., 2020) and PRRs (Davies et al., 2021; Świderská et al., 2018; Velová et al., 2018; Walsh et al., 2008). Therefore, dividing genes simply based on their assumed attribution to ‘innate’ or ‘adaptive’ immunity is unlikely to fully explain the variable evolutionary patterns we observe across immune genes. Moreover, as distinguishing alleles from different loci is difficult, MHC studies in non‐model organisms often report all variants observed per individual—so‐called MHC diversity (Eizaguirre & Lenz, 2010; Minias et al., 2018; O'Connor et al., 2016; Piertney & Oliver, 2006; Radwan et al., 2020; Richardson & Westerdahl, 2003). If different MHC gene copies are considered together, the total MHC diversity observed per population becomes markedly higher across MHC genes than for single copy genes. This makes comparisons between single‐copy genes and multilocus MHC difficult.

BOX 2 **Multicopy innate immune genes can have complex evolutionary paths**

Multigene families present a range of difficulties in evolutionary analyses, due to expansion and contraction leading to copy number variation (CNV), sequence and expression polymorphisms leading to functional differences, potential convergent evolution of paralogous gene family members, and changes in gene location that can change the rate of sequence exchange. Overall, the difficulty is knowing which gene copies can be considered orthologous for comparison between individuals and between species in evolutionary analyses.

Although there are many single‐copy genes of innate immunity, multigene families based on sequence similarity are not rare. Multigene families involved in innate immunity include natural killer cell receptors (NKRs) and their homologues that primarily recognize MHC molecules but also pathogen‐encoded peptides and decoys (Djaoud & Parham, 2020), as well as PRRs that recognize pathogen molecules (pathogen‐associated molecular patterns, PAMPs) but also self‐molecules located in the wrong context indicating stress (danger‐ or damage‐associated molecular patterns, DAMPs; Gong et al., 2020). The NKRs and their homologues include the multigene families of KIRs, LILRs, FcRs, NKG2A/CD94, NKG2D, NKR‐P1 and CD300 among others, while the PRRs include TLRs, NLRs, CLRs, scavenger receptors, AIMs, Siglecs and butyrophilins among others.

Focusing on NKRs as an example, there are molecules based on several structural families used to a greater or lesser extent in different vertebrates, with one explanation being wholesale replacement of one multigene family by another due to strong pathogen pressure (so‐called receptor switch). Even within one NKR multigene family, there can be significant variation between species and also CNV within species.

In humans, closely related multigene families of tandemly duplicated paralogs that encode cell surface proteins with immunoglobulin‐like (Ig‐like) extracellular domains are found in a cluster called the leukocyte receptor complex (LRC), including the killer Ig‐like receptors (KIRs) and leukocyte Ig‐like receptors (LILRs), as well as a single Fc receptor (FcR) and the natural cytotoxicity receptor NKp46 (Barrow & Trowsdale, 2008). Close by is the less closely‐related family of sialic acid‐binding Ig‐type lectin receptors (Siglecs; Pillai et al., 2012). KIRs are primarily expressed on NK cells, LILRs primarily on myeloid cells, the FcR on B cells, and different Siglecs on a wide variety of cells. In chickens, a similar genomic region has hundreds of chicken Ig‐like receptors (ChIR) genes, many of which encode Fc receptors, with others thought (but not proven) to be NKRs (Viertlboeck et al., 2009; Viertlboeck & Göbel, 2011). The expression of KIR genes is variegated, with clones of NK cells bearing different combinations of KIRs on their surface; along with the licensing and trained immunity of such clones which are considered analogous to education and memory of adaptive immunity (Elliott & Yokoyama, 2011; Freud et al., 2017; Netea et al., 2016). Different lineages of KIR genes are used among primates and various other mammals but are not found at all in rodents, and similar genes with very different lineages of Ig‐like domains are presumed to have the same function in other vertebrates including some fish (Guethlein et al., 2015; Wang, Belosevic, et al., 2021).

Similarly, but on another chromosome, there are multigene families of receptors with C‐type lectin extracellular domains, including Ly49, NKG2 and NKR‐P1 families which in well‐characterized mammals are found as clusters in the so‐called natural killer complex (NKC), along with others less well‐characterized (Kirkham & Carlyle, 2014). Functional Ly49 genes are not found in humans but are highly expanded in rodents in the so‐called killer cell lectin‐like receptor subfamily A (KLRA) locus, encoding cell surface proteins that bind MHC class I molecules and fulfil roughly the same functions as KIRs in humans. No KIRs are reported for rodents, perhaps an example of ‘receptor switch’, selected when an immune system becomes sufficiently poor at dealing with current pathogens. Indeed, little or no NK receptor diversity (either Ig‐like or lectin‐like) is found for some vertebrates, including naked mole rats and marine mammals (Hammond et al., 2009; Hilton et al., 2019). Most members of the NKG2 family encode cell surface proteins (NKG2A, B, C, etc) in complex with the signalling molecule CD94, recoginising non‐classical MHC class I molecules (like HLA‐E in humans and Qa1 in mice) presenting conserved signal peptides from classical MHC class I molecules as part of a molecular arms race with certain viruses; they are also as important as KIRs in licensing human NK cells during ontogeny (Guethlein et al., 2015). NKR‐P1 (also known as NK1.1, KLRB1 and CD161) genes are paired with their lectin‐like ligand genes (also known generally as CLEC2D) in the NKC of humans, mice and rats. However, a polymorphic BNK and monomorphic Blec ligand pair is found in the MHC of chickens and a similar pair on the Z sex chromosome of passerine birds, so such genes can move around in the genome, frustrating attempts to use synteny to establish orthology (Rogers & Kaufman, 2016). Although a single NKR‐P1/ligand gene pair is found in humans and chickens, the family of pairs is highly expanded in mice and rats, and the co‐evolution of these genes with decoys encoded by rat cytomegalovirus has been reported (Kirkham & Carlyle, 2014).

A similar level of complexity of structure, function, number and location of genes can be found for PRRs. However, Toll‐like receptor (TLR) genes with similar functional features can be traced through the vertebrates, in part because there are typically only around 10 genes, although some genes are duplicated or deleted in particular taxa (Liu et al., 2020). Some researchers would view such gene families (as determined by sequence and structural similarity) as behaving more like single‐copy genes in evolution, significantly simplifying their analyses (Velová et al., 2018). In contrast, there are hundreds of TLR, NLR and scavenger receptor genes found in sea urchins (Buckley & Rast, 2012), and large differences in gene copy number are found within vertebrates for the NLRs, with 20 or so in humans but thousands in zebrafish (Howe et al., 2016). With many genes that can more‐or‐less freely exchange sequence features, tracing the evolution can be difficult.

How do these changes occur? Among the many mechanisms (including mutation, gene conversion and genetic translocation), an influential concept has been the birth‐and‐death model (Nei et al., 1997), which depends on unequal crossing‐over between members of a multigene family through homologous recombination. A guiding principle is that changing from one to two copies of a gene will generally depend on non‐homologous recombination, making this a difficult and infrequent process, but once there are two genes in the same transcriptional orientation, homologous recombination allows rapid expansion to many copies but also contraction down to as few as one copy by unequal crossing‐over between chromosomes and/or deletion within a chromosome. One strategy to reduce the potential for contraction is to organize homologous genes in opposite transcriptional organization (as can be seen within the chicken MHC, Afrache et al., 2020), for which homologous recombination would lead to inversion rather than deletion.

**FIGURE 2:**
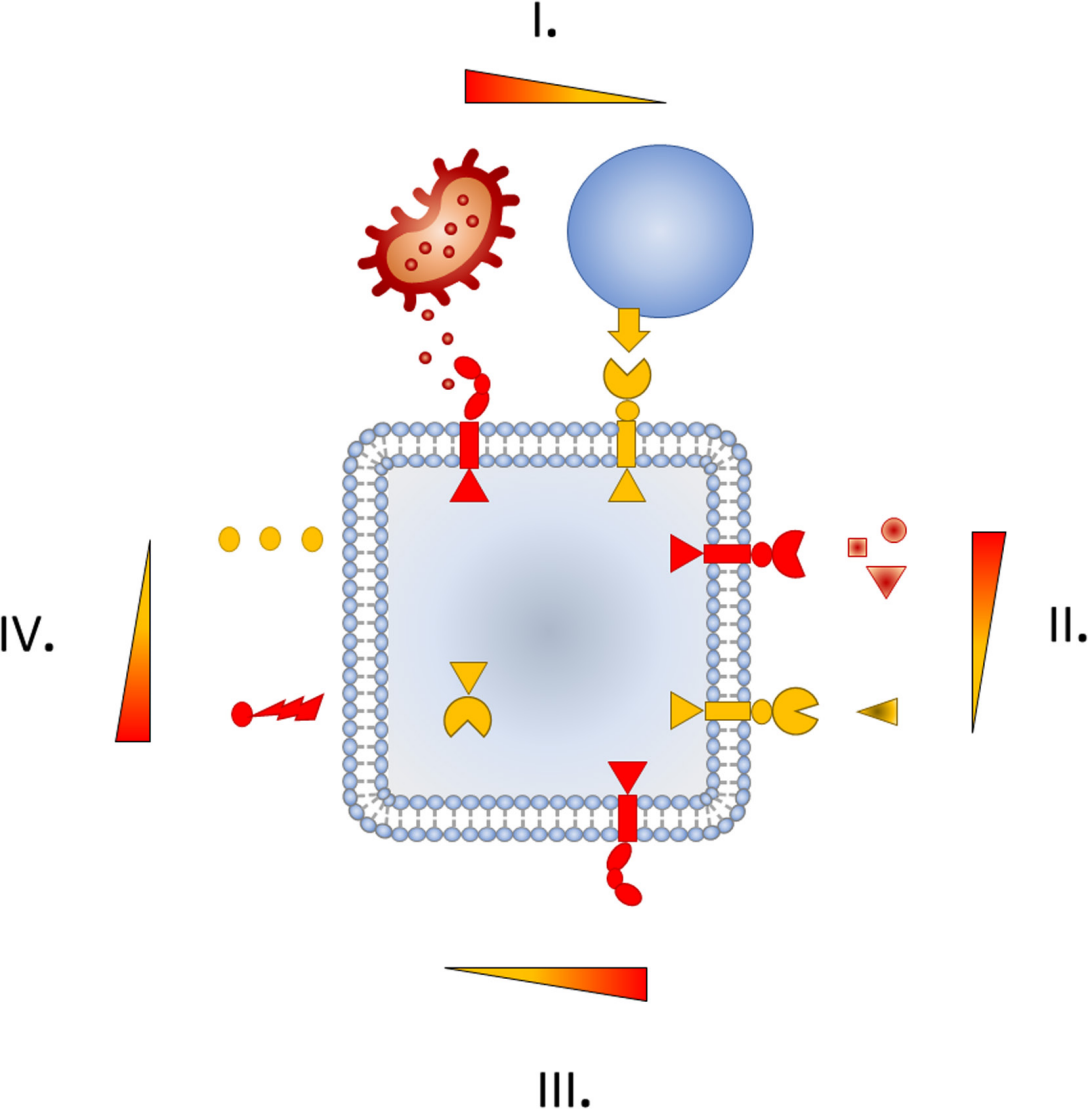
Differences in selective pressures in different immune gene sets. Current evidence suggests that groups of immune genes tend to have differing levels of genetic variation as well as strength of selection acting on them. We predict increased pathogen‐mediated natural selection (range from yellow = low to red = high) that drives increased interspecific genetic variation and intraspecific population polymorphism especially in the genes that encode proteins: (I) directly involved with pathogen structures rather than host‐self molecules; (II) involved with a high number of structurally distinct ligands rather than with structurally limited interactions; (III) expressed on the cell‐surface rather than to cytoplasm and (IV) with inducible rather than constitutive expression.

Evolutionary distinctions are also likely to exist between different functional and structural categories of immune genes. One approach could be to categorize immune genes by whether the proteins they encode function in ‘recognition’, ‘signalling’ or ‘effector’ roles (Sackton et al., 2007; Zhong et al., 2021) as clear differences can be predicted between these categories. However, multiple distinct evolutionary pressures may act even within these functional gene groups. For example, pathogen recognition can occur either directly via pathogen‐derived molecules or indirectly via host‐derived damage signals (Uematsu & Akira, 2006), leading to distinct types of evolutionary interactions acting in these two subgroups. Here we suggest combining immune genes into groups with predicted similarities in their evolutionary patterns based on their functional interactions with other molecules, forming more of a continuum of patterns, rather than a few distinct classes (Figure 2):

### 4.1 Proteins involved in direct interactions with microbial structures versus those interacting only with host self molecules

We hypothesise that host proteins that directly interface with microbial molecules will be more intensively involved in host–pathogen coevolution, thus selecting for increased functional genetic variation. This principle has been suggested for self/non‐self discrimination in immunity (Spottiswoode & Busch, 2019) and underpins investigations into MHC (Bernatchez & Landry, 2003; Hughes & Nei, 1988, 1989; Radwan et al., 2020; Spurgin & Richardson, 2010) and TLR variability (e.g. Alcaide & Edwards, 2011; Levy et al., 2020; Świderská et al., 2018). In molecules interacting only with invariant molecules derived from the host, lower variation stabilized by negative selection would be adaptive. Thus, molecules interacting with invasive pathogens should be functionally more variable, while those recognizing invariant self‐components should be conserved (Marchalonis et al., 2002). While direct support for this assumption is generally still lacking, indirect support can be taken from the well‐evidenced studies on synchronized receptor‐ligand co‐evolution (Grandchamp & Monget, 2018).

### 4.2 Proteins involved in interactions with a high number of structurally distinct ligands versus proteins involved in structurally limited interactions

Interacting with a high number of variable structures should select for broader variability (allelic diversification). Receptors interacting with their ligands often show complex structural coevolutionary patterns (Chakrabarti & Panchenko, 2010). Immune systems should evolve to optimize the balance between broad responses to heterogeneous stimulation and restricted responses only to specific stimulation. Thus, in ligand‐binding molecules, function may depend on the balance between degeneracy (i.e. the ability of one receptor to elicit a cellular response following interaction with any one of a set of structurally distinct ligands) and specificity i.e. the ability to interact productively with a single or a few ligands related in structure (Vivier & Malissen, 2005). A clear example is the hierarchy of promiscuous to fastidious MHC class I molecules in chickens and humans (Kaufman, 2018). Assuming single domains are involved, more complex structural coevolution may be required in cases of structurally variable ligands (entirely distinct molecules, Uematsu & Akira, 2006, or even heterogeneous structures of the same molecule, such as differently acylated LPS variants, Wang et al., 2015). Such patterns have been described in avian TLRs, where genes encoding receptors recognizing simple structurally invariant ligands (e.g. nucleic acids, such as in TLR3) show lower levels of positive selection than those specifically binding complex ligands (e.g. TLR2 or TLR4; Velová et al., 2018). Yet, in contrast to the co‐evolutionary patterns suggested for pathogen‐derived structures, interacting with a high number of invariant host‐self pathway components may lead to strong negative selection depleting variation (Wang et al., 2019). We predict higher adaptive genetic variation in immune recognition mechanisms based on detection/presentation of non‐self (MHC and some PRRs) than those recognizing disruption of normal integrity (PRRs) or missing self (inhibitory NK cell receptors).

### 4.3 Cell‐surface proteins and soluble proteins (outer) versus cytosolic (inner) proteins

While the inner environment of cells is strictly regulated to maintain normal physiology (Casey et al., 2010), the extracellular space often provides more changeable conditions, for example, pH (Erra Díaz et al., 2018) or heterogeneity of interacting molecules. Therefore, cytosolic components may be facing selection by less diverse stimuli than extracellular components. While little evidence is presently available to support this hypothesis, human immunogenetic research has shown that cytosolic sensors of infection, such as NOD‐like receptors (NLRs), RIG‐I‐like receptors (RLRs), Cytosolic DNA sensors (CDSs), and cytoplasmic signalling pathway members, are far less frequently associated with resistance to infectious diseases than the membrane‐bound molecular sensors, such as C‐type lectin receptors (CLRs) and TLRs (Pothlichet & Quintana‐Murci, 2013).

### 4.4 Constitutively expressed proteins versus proteins with inducible expression

In immunity, the constitutively expressed proteins typically serve in broad‐range effector mechanisms—for example, some AMPs (Zasloff, 2002)—that interfere with the growth of pathogens through mostly invariant mechanisms. This is in contrast to the proteins involved in inducible defence, where binding specificity determines the efficiency of the immune response (Eisen & Chakraborty, 2010; Marchalonis et al., 2002; Vivier & Malissen, 2005). Therefore, one could predict higher adaptive genetic variation in proteins with inducible expression compared with those constitutively expressed. Rapid evolution is especially likely in host genes that parasites might use to interfere with inducible host responses, either by blocking induction or by activating inhibitory pathways. Preliminary evidence for this has been reported by Shultz and Sackton (2019) who used a large vertebrate transcriptomic dataset to show that genes upregulated in response to pathogens are enriched for signals of positive selection. However, further research is needed to elucidate whether this pattern is unique or driven by the combination of other overlapping factors.

To effectively defend the host organisms, immune systems require a carefully optimized balance of detection and effector mechanisms exhibiting evolutionarily dynamic and conservative features. While supported by evidence from specific genes and gene families, testing the above‐mentioned predictions on whole‐genome vertebrate data is still problematic. The current structures of genome annotation databases are not suitably organized to allow automatic differentiating between the immune gene groups as defined above. Genes belonging to the same groups from the perspective of their predicted evolutionary clustering are mostly combined under different and overlapping GO terms (Kosiol et al., 2008), Reactome (Gillespie et al., 2022) and KEGG pathways (Shultz & Sackton, 2019).

## 5 HOW TO APPROACH IMMUNE GENE HETEROGENEITY IN EVOLUTIONARY STUDIES?

Studies in non‐model organisms have mainly adopted the candidate gene approach, with MHC and TLR genes being popular in a broad range of jawed vertebrates, from fish to amphibians, birds and mammals (Bagheri & Zahmatkesh, 2018; Brouwer et al., 2010; Minias & Vinkler, 2022; O'Connor et al., 2019; Wilson, 2017). Although all jawed vertebrates possess mostly overlapping core immunomes, it is evident that the final composition of immune genes and gene copy numbers can differ considerably between vertebrate classes and orders, and even within orders and families (Fiddaman et al., 2022; Meyer‐Lucht et al., 2016; Minias et al., 2019; O'Connor & Westerdahl, 2021; Těšický & Vinkler, 2015; Velová et al., 2018; Wang et al., 2012). This raises the question of how best to study patterns of selection in immune genes in non‐model organisms.

An important consideration in choosing an appropriate methodological approach is timescales. Studies that measure immune gene variation within a species are by definition done on a microevolutionary scale (Reznick & Ricklefs, 2009). Analyses of the variation that exists between species afford a macroevolutionary perspective and provide insights into large‐scale processes that shape immune gene diversity (Kolora et al., 2021; Velová et al., 2018). Past selection can be traced as genetic footprints in immune genes and the selective patterns in orthologous genes (see BOX 1) can be targeted on both micro and macroevolutionary scales (Cortázar‐Chinarro et al., 2018; Ebert & Fields, 2020; Nielsen et al., 2005; O'Connor et al., 2018; Scherman et al., 2014; Tschirren et al., 2011).

Micro and macro perspectives in evolution pinpoint slightly different evolutionary questions and have different challenges when executed. In many respects, studies conducted within species are easier than between species, because within species it is likely that all targeted immune genes will occur in all individuals. Moreover, single‐copy immune genes are much easier to compare between species than duplicated genes (paralogs), thus such innate immune genes (excluding multigene families, see BOX 2) are often easier to study on a macroevolutionary scale than those of the adaptive immune system, which are often highly duplicated (e.g. MHC class I and II genes; Fijarczyk et al., 2018; Malmstrøm et al., 2016; Westerdahl et al., 2022) or even undergo somatic recombination (e.g. TCRs and BCRs). For this reason, a focus on single‐copy genes may be appropriate to provide initial insights into a broad range of genes across species. Furthermore, evolution by loss‐of‐function should not be neglected, and gene losses have been reported in both innate and adaptive immunity (Albalat & Cañestro, 2016; Fiddaman et al., 2022; Hilton et al., 2019; Malmstrøm et al., 2016; Roth et al., 2020).

Another important point is that although often built with a similar immunome, the immune system is inherently flexible, and even closely related species may react differently to the same pathogen (Tschirren et al., 2012). This flexibility is further reflected in the ‘tolerance to infection’ strategy which some species use to reduce the negative impact of disease on host fitness (Medzhitov et al., 2012; Råberg et al., 2009), whereas others exhibit strong responses aimed at clearing the infection (resistance) despite immunopathological effects (Graham et al., 2005). Tolerance to infection has been evidenced in natural populations and verified in experimental set‐ups (Råberg et al., 2007; Savage et al., 2020; Savage & Zamudio, 2016). One example is the response to viral infection observed in some bats, whereby high basal levels of type I interferon (IFN) are partnered with low levels of inflammation, setting their response to viruses apart from many other vertebrates (Banerjee et al., 2020). Persistent virus titres reported in apparently healthy bats suggest they employ a tolerance strategy to many viruses (Irving et al., 2021). The genetic footprints observed in immune genes with respect to resistance versus tolerance to infection can be expected to differ, which should be taken into consideration in studies of immune gene evolution.

### 5.1 The study of immune genes on a microevolutionary scale (within species)

A microevolutionary perspective gives insights into how natural selection shapes immune gene variation over generations. Consequently, such studies often use population genetic approaches to understand immune gene variation within and across populations, such as examining changes in alleles frequencies over time (Charbonnel & Pemberton, 2005; Westerdahl et al., 2004) and space (Cortázar‐Chinarro et al., 2018; Gonzalez‐Quevedo et al., 2015; Strand et al., 2013). Particularly valuable insights come from long‐term studies of natural populations. For example, the study of Soay sheep (*Ovis aries*) suggests that MHC class II genes are under balancing selection, consistent with spatial and temporal heterogeneity in pathogen pressure (Huang et al., 2022). Likewise, work on the great reed warbler (*Acrocephalus arundinaceus*) reports evidence of balancing selection and high MHC class I diversity correlated with both disease resistance (Westerdahl et al., 2004, 2005, 2012) and fitness (Roved et al., 2018). Moreover, research on the Seychelles warbler (*Acrocephalus sechellensis*), shows evidence of both balancing selection at the MHC (Brouwer et al., 2010; Richardson & Westerdahl, 2003) and positive selection at the innate *TLR3* gene (Davies et al., 2021), although this latter pattern may be part of negative frequency‐dependent selection spanning decades. Balancing selection has also been documented in MHC and TLRs of wild ungulates in cross‐sectional studies (Quéméré et al., 2015, 2021).

Alleles subject to balancing selection are maintained for longer time‐intervals than alleles subject to neutral evolution. Therefore, population differentiation is expected to be less evident for immune genes subject to balancing selection than neutral markers (Borg et al., 2011; Schut et al., 2011). However, local adaptation to population‐specific pathogens can result in selective sweeps for certain advantageous immune alleles and then the population differentiation will instead be larger for certain immune genes than neutral markers (Nunes et al., 2021). Immune gene evolution must be seen in the context of population structure, immigration/emigration and environmental components, such as pathogens. Therefore we probably cannot expect to observe a consistent pattern among studies comparing population differentiation using immune genes and neutral markers unless environmental components and other constraints are taken into account (Eizaguirre & Lenz, 2010; Gonzalez‐Quevedo et al., 2015; Muirhead, 2001; Spurgin & Richardson, 2010).

### 5.2 The study of immune genes on a macroevolutionary scale (between species)

Evolutionary processes that shape immune genes within species scale up to shape the immunogenetic variation we see on a macroevolutionary level (Simons, 2002). The sequences of orthologous immune genes across species can be used to infer the strength and type of historical selection on immune genes (BOX 1); which are often found to be under positive selection (Sackton, 2020; Shultz & Sackton, 2019). Interestingly, even across closely related species orthologous immune genes may be subject to different selection pressure. A study of *TLR2* genes of two sympatric rodent populations showed that these genes are under balancing selection in bank voles, (*Myodes glareolus*), but not yellow‐necked mice (*Apodemus flavicollis)* (Tschirren et al., 2011), possibly as a result of different strategies to combat *Borrelia* infections in the two species (Zhong et al., 2021).

Studying immune gene variation across species can uncover trans‐species polymorphisms (Figueroa et al., 1988; Lenz et al., 2013). This is commonly seen in immune genes under balancing selection, with one explanatory factor being the maintenance and sharing of favourable alleles conferring protection from particular pathogens (Těšický & Vinkler, 2015). Structural genetic variation can also be revealed by studying immune genes across species (Burri et al., 2010; Minias et al., 2019; O'Connor et al., 2016, 2018; Shiina & Blancher, 2019; Velová et al., 2018; Vinkler et al., 2014). The presence or absence of particular immune genes, or even the expansion and contraction of whole immune gene families, can be indicative of their functional relevance in different species (Bainová et al., 2014; Colgan et al., 2021; Roth et al., 2020; Sackton, 2019; Sharma et al., 2020). A recent comparative study of rockfish (genus *Sebastes*) showed that particularly long‐lived species have an expanded number of immune modulatory butyrophilin genes and proposed an adaptive role for the immunosuppressive function of these genes in the long lifespans of these fish (Kolora et al., 2021). The immune system is remarkably flexible in terms of gene gain and loss (Chattopadhyay et al., 2020; Colgan et al., 2021; Divín et al., 2022; Krchlíková et al., 2023; Parham & Moffett, 2013; Solbakken et al., 2016; Velová et al., 2018). For example, gadiform fish have lost the MHC class II genes, a loss which seems to have been subsequently compensated for by an expansion of MHC class I gene copy number (Malmstrøm et al., 2016). Based on transcriptomic evidence, naked mole rats (*Heterocephalus glaber*) lack natural killer (NK) cells and show restricted diversity in the genes regulating NK cell function (Hilton et al., 2019). This is, perhaps, a consequence of their subterranean lifestyle where they potentially encounter few viruses. Furthermore, some gene losses can be compensated by evolutionary shifts in the function of the remaining genes, as observed in the mouse (*Mus musculus*) CD1 gene family, where gene‐loss compensation in ligand‐binding based on pH was observed (Dascher & Brenner, 2003).

## 6 CURRENT EVIDENCE FOR SPECIFIC PATHOGENS DRIVING IMMUNE GENE EVOLUTION

A primary challenge in the study of pathogen‐mediated immune gene evolution lies in detecting the signatures of evolution generated by specific pathogens (Spurgin & Richardson, 2010). Some immune genes are clearly hotspots of positive selection within the jawed vertebrate genome (Piasecka et al., 2018), but the specific (groups of) pathogens imposing selection on each gene or regulatory element remain largely unknown. In certain cases, alleles have been linked to disease resistance, for example, specific MHC class II alleles in Atlantic salmon (*Salmo salar*) and three‐spined stickleback (*Gasterosteus aculeatus*) confer reduced risk of bacterial or nematode infection (Bolnick et al., 2014; Dionne et al., 2007, 2009; Eizaguirre et al., 2012; Grimholt et al., 2003). However, the range of pathogens that previously interacted with these MHC genes to generate the currently observed diversity patterns is likely to have been plentiful. Although technically challenging, on‐going selection and/or recent evolution in immune genes can be measured in populations over time; such long‐term sampling approaches have been executed in some studies (Davies et al., 2021; Huang et al., 2022; Westerdahl et al., 2004). An alternative approach is screening genetic footprints of previous selection in genomes, but this may not be able to provide direct evidence for the role of any particular pathogen (Ebert & Fields, 2020). Nonetheless, see for example Souilmi et al. (2021), for putative direct connections. Genomic information can then be interpreted with bioinformatic tools to infer selection within genes and also evolution related to regulatory mutations (gene expression; Enard et al., 2014). Sequencing of genomes from historical museum samples (museomics) is also a way to view how patterns of variation at immune genes change over time (Alves et al., 2019; Irestedt et al., 2022; Krause‐Kyora et al., 2018), although again identifying the specific pathogens responsible may be difficult. In BOX 3, we highlight examples of host–pathogen interactions involving different classes of pathogens and vertebrate taxa to draw inferences about which genes and selective mechanisms may be invoked by different types of infections.

BOX 3 **Examples of host–pathogen systems revealing immune gene evolution**

**Human immunodeficiency virus**

In humans, HIV is arguably the most thoroughly studied pathogen related to host–pathogen interactions (International HIV Controllers Study et al., 2010). In genome‐wide association studies (GWAS), HIV viral load, a proxy for disease progression, has been associated with variation in two key genomic regions, the *CCR5*, used by the virus for cell entry, and the *MHC* region (McLaren et al., 2015). The latter signal could be mapped to a handful of amino acid residues in the peptide binding grooves of the MHC class I genes (International HIV Controllers Study et al., 2010; McLaren et al., 2015). Overall genomic markers account for 19–25% of the variation in viral load. Further work has shown that the MHC‐HIV association is based on both qualitative (i.e. specific peptide binding) and quantitative (i.e. number of peptides bound) aspects of peptide binding by different MHC variants (Arora et al., 2020; Chappell et al., 2015; Košmrlj et al., 2010). This recent genomic work aligns nicely with earlier work reporting MHC heterozygote advantage in slow disease progressors (Carrington et al., 1999). A number of genetic mechanisms have been shown to impact HIV disease progression, including epistasis with NK and similar myeloid receptors (Bashirova et al., 2020; Goulder & Walker, 2012; Martin et al., 2018). While this observed selection may contribute to the maintenance of high levels of MHC polymorphism in natural populations (Radwan et al., 2020), it needs to be pointed out that mortality due to the HIV infection is delayed in the lifetime, which weakens the selection efficiency of the pathogen.

**Avian viruses**

Selection of avian immune genes by viral diseases is exemplified by strong associations between the chicken MHC class I region and Marek's disease virus (Briles et al., 1977). Presence and expression of specific MHC class I haplotypes at the B locus, such as *B* ^21^, confer resistance, while other haplotypes (*B* ^13^ or *B* ^19^) are associated with susceptibility. In the case of avian influenza A viruses, chickens with homozygous *B* ^21^ haplotype had 100% survival rate, while those homozygous for the *B* ^12^ or *B* ^13^ haplotypes suffered 100% mortality (Boonyanuwat et al., 2006). This evidence documents the importance of MHC alleles binding broad ranges of different peptides (*B* ^21^) in resistance to viral infections, compared with specialists binding more narrow peptide repertoires (Chappell et al., 2015). On the other hand, specialized alleles (*B* ^12^) may provide advantage to specific pathogens, such as the Rous sarcoma virus (Hofmann et al., 2003; Wallny et al., 2006). Genomic evidence from one of the key natural reservoirs for influenza A, the Mallard duck (*Anas platyrhynchos*) indicates that β‐defensin and butyrophilin‐like gene families also play key roles in host responses to avian influenza (Huang et al., 2013). Likewise, using genomic approaches novel genes associated with *Avipoxvirus*, involved in cellular stress signalling and immune responses, were identified across divergent island populations of Berthelot's pipit (*Anthus berthelotii*; Sheppard et al., 2022).

***Ranavirus* in ectotherms**

The genus *Ranavirus* includes eight species of viruses that infect many ectothermic vertebrates including, to date, 34 reptile, 27 fish and 63 amphibian genera (Brunner et al., 2021). While host species and developmental stages vary in susceptibility, ranaviruses have played a major role in mass mortalities of multiple species (e.g. Geng et al., 2010; Johnson et al., 2008; Price et al., 2014). Although still poorly understood, the ranavirus infection triggers broad‐scale immune responses (reviewed in Grayfer et al., 2015) involving type I and III interferon (IFN), tumour necrosis factor‐alpha (TNFA), interleukin 1‐beta (IL1B), myxovirus resistance protein 1 (Mx1) and TLRs. Humoral and cellular immunity are also activated with ranavirus‐specific immunoglobulins (IgY) and T cells ranging from cytotoxic CD8 T cells to an invariant T cell population (CD8 ^–^ /CD4 ^–^) dependent on XNC10 (a nonclassical MHC class Ib gene). However, although the repertoire of potential targets of ranavirus‐mediated selection among the immune genes is large, solid evidence is still scarce. Some immune genes seem to be differentially expressed between frog populations with and without ranaviral disease history (Campbell et al., 2018) and some MHC class I and II supertypes may be associated with resistance to ranavirus infection (Savage et al., 2019; Teacher et al., 2009).

**Bacterial infection in mammals**

*Mycobacterium avium* subsp. *paratuberculosis* (MAP) is the agent of Johne's disease in ruminants. Shared susceptibility to MAP in domesticated and wild populations makes ungulates an excellent model for studying the adaptive evolution of immune genes (Chebii et al., 2021; Griffin, 2014; Jolles et al., 2015; Ren et al., 2021). The role of *TLR2* and other *TLRs* in ungulate defence against MAP has been documented (Fisher et al., 2011), and a GWAS identified a number of candidate genes for MAP resistance, including *NLRP3*, *IFi47*, *TRIM41*, *TNFRSF18* and *TNFRSF4* (Mallikarjunappa et al., 2018). While in total more than 200 bovine genes were suggested to contribute to MAP resistance, functional validation is still mostly missing (Mallikarjunappa et al., 2021). Analogous genetic mapping efforts in humans, focusing on *M. tuberculosis*, identified a number of resistance factors against the disease, including PRRs (Dubé et al., 2021). Wild rodents are the main reservoir of many bacteria (Ostfeld, 2012), and in a wild population of bank voles (*Myodes glareolus*), one of the main *TLR2* haplotype clusters was associated with resistance to tick‐transmitted *Borrelia afzelii* (Tschirren et al., 2013). Given the patterns of distribution of the *TLR2* polymorphism in vole populations across Europe, the *TLR2* variation appears to be maintained long‐term (Morger et al., 2015). Furthermore, recent research in MHC class II alleles (DQB) unravelled complex host genotype‐by‐pathogen genotype interactions between the voles and *Borrelia* strains (Råberg et al., 2022; Scherman et al., 2021).

***Mycoplasma gallisepticum* in birds**

In the 1990s, the bacterial pathogen *Mycoplasma gallisepticum* (MG) spilled over from domestic poultry into the house finch (*Haemorhous mexicanus*) (Ley et al., 1996; Luttrell et al., 1998). In two decades, MG spread across North America (Hochachka et al., 2013; Staley et al., 2018), causing up to a 60% population decrease (Hochachka & Dhondt, 2000) and inducing strong selection. Comparing an MG‐naïve population of house finches to one with a history of MG endemism, Zhang et al. (2014) identified two genes that showed positive selection: the *HSPBAP1* gene associated with heat‐shock and a T‐cell precursor gene *THYMIS*. Moreover, low MHC class II diversity was associated with increased disease severity (conjunctivitis) during MG infection (Hawley & Fleischer, 2012). Furthermore, birds with a particular HSP90‐alpha genotype had lower pathogen loads (Backstrom et al., 2013). Selection driven by MG also shapes expression patterns in various immune genes, including MHC class II‐associated invariant chain and neutrophil cytosolic factor 4 (*NCF4*; Bonneaud et al., 2011). MG evolves to promote conjunctival inflammation, possibly to facilitate its transmission, upregulating expression of pro‐inflammatory cytokines, including *IL1B* and *IL6* (Vinkler et al., 2018). Interestingly, the house finches evolve to counteradapt, as evidenced by *IL1B* expression only being elevated during MG infection in birds from an MG‐naïve population, but not those with a long history of MG endemism (Adelman et al., 2013). This suggests that there may be selection for tolerance to MG infection in house finches.

***Batrachochytrium* in amphibians**

Chytridiomycosis is a fungal skin infection caused by *Batrachochytrium dedrobatidis* (Bd) that emerged in amphibian hosts worldwide over the past half‐century. Bd infection has caused the greatest pathogen‐driven loss of biodiversity ever recorded (Scheele et al., 2019), suggesting that Bd imposes potent selective pressure on host immune systems. Indeed, positive selection has been revealed in AMP and MHC genes in affected hosts (Kosch et al., 2016, 2017; Woodhams et al., 2010), transcriptome‐wide gene expression changes in response to Bd infection have been described (Ellison, Savage, et al., 2014; Ellison, Tunstall, et al., 2014 Eskew et al., 2018; Poorten & Rosenblum, 2016; Ribas et al., 2009; Rosenblum et al., 2012; Savage et al., 2020), and AMP and MHC immune variation associated with host Bd susceptibility has been identified (Bataille et al., 2015; Savage et al., 2018; Woodhams et al., 2007). While most of these studies are unable to show that Bd is the definitive cause of immunogenetic change, extensive work on MHC evolution provides the most compelling evidence that Bd has prompted rapid immunogenetic adaptation. For example, studies of the lowland leopard frog (*Rana yavapaiensis*) have demonstrated that an MHC class II variant (allele Q) arose under recent positive selection, is associated with Bd survival and only occurs in high frequency in natural populations that are Bd tolerant (Savage & Zamudio, 2011, 2016). Likewise, alpine tree frogs (*Litoria verreauxii alpina*) from a population with a long history of Bd exposure significantly upregulated multiple immune genes (including MHC) relative to one that was only recently exposed to Bd (Grogan et al., 2018), indicating evolved gene expression shifts after multiple generations of Bd selective pressure. Recently MHC class II alleles in the common toad (*Bufo bufo*) have been shown to associate with strain‐specific differences in survival after experimental Bd infection (Cortázar‐Chinarro et al., 2022).

1 ***Trypanosomes* in cattle**

Evidence of *Trypanosoma*‐induced immune adaptation comes from studying the migration of cattle across Africa, estimated to start at 5000 BP (Gautier et al., 2009; Mwai et al., 2015). Cattle were subject to new selection factors, including trypanosomiasis, as they spread across novel tropical environments, which drove changes in the gene pools of domestic cattle (Smetko et al., 2015). Whole genome Bayesian scans of cattle populations in west Africa have identified footprints of adaptive selection comprising 53 genomic regions and 42 candidate genes, including strong balancing selection on the MHC (Gautier et al., 2009). Another identified candidate gene, *TICAM1* codes for a TLR adapter and thus plays a key role in innate immunity. Similarly, natural selection appears to have shaped the gene encoding chemokine receptor CXCR4, a modulator of T‐cell responses, which coincides with the previously identified QTL for trypanosome tolerance (Hanotte et al., 2003). Finally, the receptor for anthrax toxin (*ANTRXR2*) has been identified among the positively selected genes (Gautier et al., 2009). This example illustrates the importance of reconstructing the historical population genetic structure for evidencing the contribution of genes to host adaptation against newly encountered pathogens.

2 **Transmissible cancer in Tasmanian devils**

Transmissible cancers are a rare type of pathogen where a single cancer evolves to become infectious and spreads through a population. Devil facial tumour disease (DFTD), a transmissible cancer of the Tasmanian devil (*Sarcophilus harrisii*), has had a mortality rate close to 100% in adult devils (Margres et al., 2020; Wright et al., 2017), which has stimulated strong selection within the population. Selection can occur despite such a high mortality rate as DFTD can take up to 1 to 2 years to kill the host. So devils that breed younger, invest more into a single breeding season or tolerate the tumours for longer periods are able to contribute more to the next generation (Lachish et al., 2009; Wells et al., 2017). Within a few generations there have been allele frequency changes (detected using SNPs) in genomic areas surrounding immune‐ and cancer‐related genes (including CD146, THY1, CRBN; Epstein et al., 2016; Hubert et al., 2018; Stahlke et al., 2021), a reduction in alleles associated with abiotic local adaptation (Fraik et al., 2020) and even changes in gene expression with DFTD infection (immunoglobulins, NKG2D, CD16; Raven et al., 2022; Ujvari et al., 2016). The *PAX3* gene has been associated with documented tumour regressions, possibly involved in a regulatory pathway, slowing tumour growth (Wright et al., 2017). In addition, the expression of *RASL11A* in the tumours, stimulating the Rat sarcoma pathway, has been connected to documented tumour regressions (Margres et al., 2020). However, more recently the rapid evolution has been questioned due to allele frequencies at putative DFTD‐associate loci being similar in wild devils exposed to DFTD and those from the insurance population which have had little or no exposure to DFTD (Farquharson et al., 2022).

3 **Cestodes in three‐spined sticklebacks**

Three‐spined sticklebacks (*Gasterosteus aculeatus*) are frequently infected by a cestode, *Schistocephalus solidus*. This tapeworm is known to affect various host traits, including behaviour (Berger et al., 2021), and impact host fitness (Barber et al., 2008; Heins & Baker, 2008). Stickleback populations have been shown to exhibit varying resistance against *S. solidus*, possibly in response to differential prevalence of the parasite in their natural habitats (Piecyk et al., 2019; Weber et al., 2017). In some populations, resistant fish mount an intensive fibrosis response that traps the parasite in a cyst. An experimental cross between a high‐prevalence (tolerant) and low‐prevalence (resistant) stickleback population identified two major quantitative trait loci (QTL) (Weber et al., 2022). Selection screens within these QTL identified that the *SPI1* gene, a major regulatory switch controlling fibroblast polarization to generate fibrosis (Watt et al., 2021; Wohlfahrt et al., 2019), is up‐regulated in more fibrotic fish and that knock‐outs of *SPI1B* exhibit altered fibrosis (Fuess et al., 2021). The other major QTL contributes to both inflammation (respiratory burst) and cestode growth suppression above and beyond the effect of fibrosis, possibly involving the immune genes *STAT6* and *HNF4A*. Thus, evidence points to selection acting on multiple genes (NIRGs as well as core immunome genes), contributing to the suppression of cestode growth and viability in sticklebacks. A specific role for MHC variability in *S. solidus* infection remains debated (Kurtz et al., 2004; Natsopoulou et al., 2012; Peng et al., 2021; Stutz & Bolnick, 2017).

### 6.1 Complex patterns of pathogen‐mediated natural selection acting on immune genes

The examples in BOX 3, along with many others in the literature, highlight that disease resistance is often a polygenic trait, including genes of the core and peripheral immunome as well as NIRGs. While a single pathogen can have a strong effect on host fitness and produce detectable host evolution, an array of pathogens will contribute to the total selective pressure experienced by a host. Simultaneous exposure to multiple pathogens (probably the norm in wild populations) can lead to complex patterns of selection that might be difficult to disentangle (Alizon & van Baalen, 2008; Cattadori et al., 2008; Garcia‐Longoria et al., 2022; Schmid‐Hempel, 2011). Furthermore, different pathogens may act on different spatial–temporal scales or illicit different mechanisms of selection, again complicating signatures of immunogenetic evolution (Spurgin & Richardson, 2010). One aspect in which pathogens differ from each other is the composition of their immunity agonists, from PRR ligands to antigens presented by MHC molecules. A recent analysis showed that most human pathogens share few antigen peptides, suggesting that different pathogens will select for different MHC variants with distinct peptide binding properties, providing a possible explanation for high allelic diversity at the MHC genes (Özer & Lenz, 2021). Yet, some MHC alleles bind a wide variety of peptides and confer resistance to many unrelated pathogens (Kaufman, 2018). Differences among pathogens (both species and strains) and the modes of recognition by host receptors are the basis of any hypothesis for pathogen‐mediated balancing selection, and a better understanding of these differences will be a key to understanding the evolution of immune genes at large.

## 7 FUTURE DIRECTIONS

Until recently, evolutionary patterns in immune genes in non‐model organisms were predominantly investigated in a limited number of gene sets, that is, MHCs and TLRs. However, genomic research has found evidence that a broader range of immune genes involved in the immunome experience rapid evolution, including other components of adaptive immunity, inflammatory pathways and complement (Kosiol et al., 2008). Future research needs to take a broader scope and include the peripheral immunome and NIRGs. With improved sequencing technology and dedicated analysis pipelines it now becomes feasible to investigate ultra‐diverse immune receptor (TCR, BCR) repertoires in non‐model species (Migalska et al., 2018) as well as gene expression in different tissues and different cell types (Scalf et al., 2019; Sudmant et al., 2015).

With the pace at which high‐quality genomes are being produced through key consortia (e.g. Tree of Life and Vertebrate Genome Project), the availability of species‐specific reference genomes should not be a limitation for much longer. However, research using long‐read sequencing techniques shows that classical short‐read reference genomes alone are not sufficient to grasp the structural genetic variation in complex immune gene families (Vekemans et al., 2021). Recent methodological advances allow large population screenings, comparative analyses of populations or even investigation of allele frequency shifts based on historical samples. Yet, availability of the genomic information alone is not enough to promote our understanding of the evolutionary processes diversifying immune responses in animals. Improved genome annotations and a better understanding of gene function across species boundaries are also imperative. In most non‐model animal species evolutionary researchers have struggled with a lack of substantial genome annotations. This challenge can be partially overcome using annotations from related model organisms, but to a certain extent experimental functional annotation may still be required in the species of interest. The need for such testing depends heavily on the gene investigated. While in gene orthologs functional conservatism can be predicted, in cases of frequent paralogous genes, such as MHC, only limited predictions can be made. Moreover, careful considerations must be also taken when comparing MHC genes and MHC genetic diversity among distantly related genera/families since both phylogenetic history of MHC class I and MHC class II genes and their expression differ considerably among species (Burri et al., 2010; Chen et al., 2015; Kaufman et al., 1999; Shiina et al., 2017; Westerdahl et al., 2022). These limitations have to be considered when interpreting results and conclusions should be correspondingly cautious.

A greater level of functional testing, consistent with that undertaken by immunologists in classical model species, will be needed to verify the effects of individual adaptive variation that has emerged through host‐pathogen coevolution. In silico predictions (Follin et al., 2013; Králová et al., 2018; Těšický et al., 2020) and structural analysis (Chappell et al., 2015; Koch et al., 2007) can guide this functional testing (Fiddaman et al., 2022; Hyland et al., 2021; Levy et al., 2020) to make these efforts feasible. Although such functional approaches are beyond the scope of the present review, we argue they require future attention.

## 8 CONCLUSION

Evolutionary immunology is a field that emerged from comparative immunology (Flies & Wild Comparative Immunology Consortium, 2020) and evolutionary ecology (Sheldon & Verhulst, 1996). Yet, advances in this discipline are hindered by a lack of conceptual unity between researchers with backgrounds in different fields, making it challenging to form collaborative networks. The interdisciplinary discussion opened at the ESEB 2021 Online Satellite Symposium: ‘Molecular evolution of the vertebrate immune system, from the lab to natural populations’ took the community beyond reviewing the state‐of‐the‐art in evolutionary immunology. To help expose, understand and reconcile the differing evidence and insights gained from different disciplines, we discussed key concepts and major shifts in research topics and approaches. One main conclusion is that evolutionary genetics needs to be integrated with other types of data explaining the immunological mechanisms involved in the evolution of host immune defence. Improved interpretation of results through such integrated analyses is key to advancing this field. This is best achieved through the collaborative involvement of scientists with different backgrounds, skills and perspectives; essentially developing a true interdisciplinary basis for the field. As a community, we feel the initiated strategy of interdisciplinary meetings between evolutionary biologists, immunologists, bioinformaticians, ecologists, zoologists, microbiologists, parasitologists and veterinary scientists is highly conducive to develop evolutionary immunology as an interdisciplinary research field. We hope this collaborative review stimulates discussion and may form the foundation for novel studies and collaborations in the future.

## AUTHOR CONTRIBUTIONS

**Michal Vinkler:** Conceptualization (lead); visualization (equal); writing – original draft (lead); writing – review and editing (lead). **Steven R. Fiddaman:** Conceptualization (equal); visualization (equal); writing – original draft (equal); writing – review and editing (equal). **Martin Těšický:** Conceptualization (equal); writing – original draft (equal); writing – review and editing (equal). **Emily A. O'Connor:** Conceptualization (equal); writing – original draft (equal); writing – review and editing (equal). **Anna Savage:** Conceptualization (equal); writing – original draft (equal); writing – review and editing (equal). **Tobias L. Lenz:** Conceptualization (equal); writing – original draft (equal); writing – review and editing (equal). **Adrian L. Smith:** Conceptualization (equal); writing – original draft (equal); writing – review and editing (equal). **Jim Kaufman:** Conceptualization (equal); writing – original draft (equal); writing – review and editing (equal). **Daniel I. Bolnick:** Conceptualization (equal); writing – original draft (equal); writing – review and editing (supporting). **Charli Davies:** Conceptualization (equal); writing – original draft (equal); writing – review and editing (supporting). **Neira Dedić:** Conceptualization (equal); writing – original draft (equal). **Andrew S. Flies:** Conceptualization (equal); writing – original draft (equal); writing – review and editing (supporting). **Mercedes Gómez Samblás:** Conceptualization (equal); writing – original draft (equal). **Amberleigh E. Henschen:** Conceptualization (equal); writing – original draft (equal). **Karel Novák:** Conceptualization (equal); writing – original draft (equal); writing – review and editing (supporting). **Gemma Palomar:** Conceptualization (equal); writing – original draft (equal). **Nynke Raven:** Conceptualization (equal); writing – original draft (equal). **Kalifa Samaké:** Conceptualization (equal); writing – original draft (equal). **Joel Slade:** Conceptualization (equal); writing – original draft (equal). **Nithya KuttiyarthuVeetil:** Conceptualization (equal); writing – original draft (equal); writing – review and editing (supporting). **Eleni Voukali:** Conceptualization (equal); writing – original draft (equal). **Jacob Höglund:** Conceptualization (equal); writing – review and editing (equal). **David Richardson:** Conceptualization (equal); writing – original draft (equal); writing – review and editing (equal). **Helena Westerdahl:** Conceptualization (lead); writing – original draft (lead); writing – review and editing (lead).

## CONFLICT OF INTEREST STATEMENT

The authors have no conflict of interest to declare.

### PEER REVIEW

The peer review history for this article is available at https://www.webofscience.com/api/gateway/wos/peer‐review/10.1111/jeb.14181.

## Data Availability

N/A
